# Association between plasma cadmium and renal stone prevalence in adults in rural areas of Guangxi, China: a case–control study

**DOI:** 10.1186/s12882-022-02945-x

**Published:** 2022-09-28

**Authors:** You Li, Kailian He, Liang Cao, Xu Tang, Ruoyu Gou, Tingyu Luo, Song Xiao, Ziqi Chen, Tingjun Li, Jian Qin, Zhiyong Zhang, Jiansheng Cai

**Affiliations:** 1 Department of Environmental Health and Occupational Medicine, School of Public Health, Guilin Medical University, Lingui District, No. 1 Zhiyuan Road, Guilin, 541199 Guangxi China; 2 Guangxi Health Commission Key Laboratory of Entire Lifecycle Health and Care (Guilin Medical University ), Lingui District, No. 1 Zhiyuan Road, Guilin, 541199 Guangxi China; 3 Department of Experimental Teaching Center, School of Public Health, Guilin Medical University, Lingui District, No. 1 Zhiyuan Road, Guilin, 541199 Guangxi China; 4 Department of Environmental and Occupational Health, School of Public Health, Guangxi Medical University, Shuangyong Road No.22Guangxi province, Nanning, 530021 People’s Republic of China; 5 Guangxi Key Laboratory of Tumor Immunology and Microenvironmental Regulation, Guilin Medical University, Lingui District, No. 1 Zhiyuan Road, Guilin, 541199 Guangxi China

**Keywords:** Cadmium, Kidney stones, Plasma, Case–control study

## Abstract

**Background:**

Kidney stones have become a worldwide public health problem. The purpose of this research is to study the relationship between plasma cadmium level and the prevalence of kidney stones in an adult population.

**Methods:**

The data of this study were based on a current survey conducted from December 2018 to November 2019 in Gongcheng Yao Autonomous County, Guangxi, China. A total of 940 study subjects of the same sex and age (within 2 years of each other) according to 1:1 matching were selected for a case–control study. The diagnosis of kidney stones was based on the presence of strong light spots, patches, clusters, or bands within the renal sinus region, followed by an echo-free bundle of acoustic images. Plasma metal elements were determined by the metal plasma method. The relationship between plasma cadmium concentration and the prevalence of kidney stones was assessed using logistic regression and restricted cubic spline regression.

**Results:**

The crude ratio for kidney stones in the highest quartile of plasma cadmium was 1.164 (95% CI, 1.121 to 2.324) compared with the lowest quartile. A positive correlation was found between the two (P for trend = 0.039). After adjusting for potential confounders, the ratio of plasma cadmium to kidney stones in the highest quartile was 1.606 (95% CI, 1.100 to 2.344) compared with the lowest quartile, and the findings remained unchanged.

**Conclusion:**

The odds of kidney stones in adults increased with increasing plasma cadmium exposure, and high plasma cadmium may be a risk factor for kidney stones.

## Introduction

Kidney stones are among the common diseases of the urinary system. Their formation starts with the deposition of minerals in the renal calyces and renal pelvis. Some patients present with renal colic, hematuria, urinary tract obstruction, and urinary tract infection [1]. In severe cases, kidney failure may occur, thereby endangering life and health. The incidence of kidney stones is on the rise worldwide [2]. The prevalence of kidney stones in the United States is 8.8% [3]; this is 9.1% for Saudi Arabia [4]. The prevalence of kidney stones is 3.6% in Northern China [5]. The total prevalence of kidney stones in China is 7.54% [6]. In addition, the recurrence rate of kidney stones is also on the rise, with 50% of patients experiencing recurrence within 5 years [7, 8]. It has become a public health problem with a significant impact on human health and society [9]. The formation of kidney stones is associated with various factors, such as metabolism, diet, genetics, environment, and underlying diseases [10–12]. Cadmium is a toxic heavy metal and a non-essential element for the human body that is widely distributed in nature; it has high toxicity, is difficult to degrade, has strong accumulation characteristics, and has a latent period of up to 10–30 years [13]. The kidney is the most important accumulation site of cadmium and is also the most sensitive to cadmium toxicity and the most easily damaged effector organ under cadmium exposure  [13, 14]. The association between plasma cadmium and kidney stones was explored by measuring plasma cadmium levels in adults in Guangxi, China to provide some theoretical basis for its prevention and control.

## Objects and methods

### Research subjects

This study was conducted from December 2018 to November 2019 to obtain information on population-based physical examinations and baseline questionnaires in Gongcheng County, Guangxi. The inclusion criteria of study subjects were as follows: ① local resident population; ② patients with renal stones diagnosed by transabdominal color Doppler ultrasound: strong spots, patches, clusters, or bands of light within the renal sinus region, followed by an echoless bundle of sound; ③ age ≥ 30 years old; ④ able to complete all physical examinations and baseline questionnaires; and ⑤ no occupational metal exposure. The exclusion criteria were as follows: ① cannot cooperate in completing all physical examinations or questionnaires due to mental illness or lack of patience; ② incomplete questionnaire information; ③ have not completed the relevant medical examination (lack of height, weight, blood pressure, and others); ④ with abnormal metal values (defined as three times the 99th percentile); and ⑤ with past or present metal contact occupations. For the case–control study, participants with the same sex and age (within 2 years of each other) were selected according to 1:1 matching. A total of 940 study subjects (740 males and 200 females)were eventually included. The majority of these studies were conducted on farmers.

### Study population

The study participants were recruited with the help of staff from the local health center and resident village cadres. Questionnaires were administered and basic information was collected through a face-to-face approach by uniformly trained investigators using self-designed questionnaires. The questionnaire collected demographic information, such as gender, age, ethnicity, education level, occupation, and smoking and drinking statuses of the study subjects. Along with the questionnaire, the height, weight, and blood pressure of the study subjects were collected by professional check. Blood pressure was measured using a mercury column sphygmomanometer. Body mass index (BMI) = weight (kg)/height^2^ (m^2^) was calculated based on the weight height data. Fasting venous blood was collected uniformly by the nurse of the medical examination center of the local township health center from the study subjects after at least 12 h of fasting. Blood samples were refrigerated and sent to the laboratory of the Gongcheng Yao Autonomous County People's Hospital, where serum total cholesterol (TC), triglycerides (TG), high-density lipoprotein cholesterol (HDL-C), low-density lipoprotein cholesterol (LDL-C), creatinine (CREA), urea (UREA), and uric acid (UA) were measured by professionals using a fully automated clinical chemistry analyzer (Hitachi 7600–020, Kyoto, Japan).

### Assessment of kidney stones

All subjects included in this study had undergone abdominal ultrasound examination. The diagnosis of kidney stones was based on the presence of strong light spots, patches, clusters, or bands within the renal sinus region, followed by an echo-free bundle of acoustic images.

### Determination of plasma metals

Blood samples from the study subjects were centrifuged at 4 °C for 10 min. Plasma samples were stored in 1.5 ml centrifuge tubes (Eppendorf, Germany) and kept in an ultra-low temperature refrigerator at -80 °C for backup. Metal element concentrations were determined using an inductively coupled plasma mass spectrometer (Thermo Fisher scientific iCAPRQ01408), as follows. Sampling system nebulizer chamber and nebulizer were immersed in 10% HNO_3_ for more than 24 h). Sampling cone and interceptor cone were cleaned in 1% HNO_3_, rinsed with ultrapure water, and dried on standby). Other instruments included ultrapure water preparation apparatus and 5 mL Ep tubes (Eppendorf Germany). The plasma samples were thawed and vortex-shaken to 100 μL, diluted 20 times with 1.9 mL of 1% HNO_3_, and then injected directly into the sample for determination. The samples and reagent blanks were determined using the assay’s standard series of operations, and the analysis was repeated thrice for each sample. The average value was taken to obtain the concentration of the measured metal element (μg/L). Quality control included the following: ClinChek® human plasma controls for trace elements Level 1 (No. 8883) and Level 2 (No. 8884); and Recipe Chemicals. The recoveries of the standard additions for each element ranged from 80.16%–114.65%. The detection limits of plasma metals ranged from 0.002 μg/L to 0.071 μg/L. All plasma metals detected in this study were above the detection limits.

### Ethical considerations

Our research protocol was approved by the Ethics Committee of Guilin Medical University (No.20180702–3).The study purpose was explained to potential participants who met the inclusion criteria, and written informed consent was obtained from all participants.

### Statistical analysis

The questionnaire data were double entered in parallel using EpiData v3.1. Continuous variables were expressed as mean ± standard deviation (± s). The normality of continuous variables was tested by Kolmogorov–Smirnov test. Data conforming to normal distribution were compared between groups using the independent sample t-test. The categorical variables were expressed as frequencies (percentages) and analyzed using chi-square tests. After performing univariate analysis, we further explored the relationship between creatinine, urea, uric acid, plasma cadmium, plasma strontium, plasma barium, and renal stones using binary logistic analysis. Plasma cadmium values were divided into four categories according to interquartile distribution, as follows: ≤ 0.12 μg/L; 0.13–0.20 μg/L; 0.21–0.28 μg/L; and ≥ 0.29 μg/L. The relationship between plasma cadmium and kidney stones was assessed using the odds ratio (OR) and 95% confidence interval (95% CI). The 95% confidence interval was calculated using logistic regression with the lowest quartile as the reference category. The choice of covariates was based on a similar previously published study [15–17].

Three models were used for multivariate analysis, as follows: Model 1 crude model; Model 2 included age (continuous data), gender (male, female), BMI (continuous data), nation (Han/Yao/Zhuang and others), marital status (unmarried and others/married), occupational (farmers/eles), education level (below junior high school/junior high school and above), SBP (continuous data), DBP (continuous data), smoking status (yes/no), and drinking status (yes/no); and Model 3 added creatinine (continuous data), urea (continuous data), and uric acid (continuous data) on the basis of model 2. Linear trend tests were performed using logistic regression analysis. To reduce skew, metal cadmium values were Log10 transformed. Subsequently, we used restricted cubic spline regression to plot the dose–response relationship between plasma cadmium concentrations and the prevalence of kidney stones using three nodes of the transformed plasma cadmium values (-1.05; -0.71; -0.36) in the R software. Statistical analysis of the above was performed using SPSS version 23.0 and R4.1.2 statistical packages, and a two-sided test *p*-value ≤ 0.05 was considered statistically significant.

## Results

Basic characteristics of research subjects. A total of 940 people who participated in the completion of health check-ups, testing of various biochemical indicators and filling out questionnaire information were selected for inclusion in this study. Among these, 740 (78.72%) were males and 200 (21.28%) were females. They were aged between 30 and 85 years old, with a mean age of 57.83 ± 11.83 years old. The results of univariate analysis showed that a statistical association existed between the kidney stone group and the non-kidney stone group in blood creatinine (CR), urea (UREA), uric acid (UA), plasma strontium, plasma antimony, plasma barium, and plasma cadmium with the occurrence of kidney stones, and these differences were statistically significant (*P* < 0.05). No statistical association was found between them and other indicators (Table 1).

**Table 1:** The relationship between kidney stones and related factors

Characteristics	Kidney stone status(%)		^2^/t	*P*
	Kidney stone group	Control group		
Gender, n (%)			-	-
Male	370(78.72)	370(78.72)		
Female	100(21.28)	100(21.28)		
Age(y)	57.83 ± 11.83	57.83 ± 11.83	-	-
30 ~ 49	42.69 ± 5.43	42.69 ± 5.43		
50 ~ 69	59.33 ± 5.81	59.33 ± 5.81		
≥ 70	74.53 ± 4.01	74.53 ± 4.01		
Nation, n (%)			0.328	0.849
Han	72(15.32)	77(16.38)		
Yao	378(80.43)	371(78.94)		
Zhuang and others	20(4.25)	22(4.68)		
Marital status, n (%)			0.008	1.000
Unmarried and others	70(14.89)	71(15.11)		
Married	400(85.11)	399(84.89)		
Occupational, n (%)			1.483	0.264
Farmers	421(89.57)	409(87.02)		
Others	49(10.43)	61(12.98)		
Education level, n (%)			1.235	0.296
Below junior high school	242(51.49)	259(55.11)		
Junior high school and above	228(48.51)	211(44.89)		
Smoking, n (%)	78(40.80)	69(35.00)	0.073	0.787
Drinking, n (%)	89(49.50)	96(48.70)	1.097	0.295
BMI (kg/m^2^)	22.90 ± 3.18	22.90 ± 3.39	0.005	0.996
SBP (mmHg)	132.24 ± 24.19	133.64 ± 24.84	0.899	0.369
DBP (mmHg)	81.73 ± 14.59	82.23 ± 13.98	0.541	0.589
TG (mmol/L)	1.55 ± 1.91	1.45 ± 1.42	-0.857	0.392
HDL-C (mmol/L)	1.71 ± 0.44	1.73 ± 0.43	0.735	0.463
LDL-C (mmol/L)	3.39 ± 0.99	3.40 ± 1.03	0.092	0.927
TC (mmol/L)	5.53 ± 1.19	5.48 ± 1.09	-0.708	0.479
CREA(μmol/L)	85.59 ± 27.01	77.16 ± 21.08	-5.334	< 0.001
UREA(mmol/L)	5.96 ± 1.80	5.70 ± 1.60	-2.316	0.021
UA (mmol/L)	360.06 ± 106.35	331.05 ± 87.98	-4.557	< 0.001
Plasma magnesium (μg/L)	18,627.35 ± 3459.77	18,521.67 ± 3270.67	-0.714	0.475
Plasma calcium (μg/L)	71,669.19 ± 11,283.51	70,601.65 ± 10,281.93	-1.427	0.154
Plasma manganese (μg/L)	3.09 ± 8.68	2.61 ± 1.68	-1.348	0.178
Plasma iron (μg/L)	1182.81 ± 468.38	1210.87 ± 485.07	0.971	0.332
Plasma Copper (μg/L)	900.18 ± 200.98	897.15 ± 203.91	-0.579	0.562
Plasma zinc (μg/L)	4115.12 ± 5427.17	3849.89 ± 5460.87	-1.260	0.208
Plasma strontium (μg/L)	31.06 ± 11.13	29.24 ± 11.35	-2.841	0.005
Plasma antimony (μg/L)	5.76 ± 12.56	3.79 ± 8.27	-2.061	0.040
Plasma barium (μg/L)	28.70 ± 15.13	26.81 ± 12.73	-1.960	0.050
Plasma lead (μg/L)	9.50 ± 17.33	8.47 ± 11.46	-1.003	0.316
Plasma cadmium (μg/L)	0.26 ± 0.21	0.24 ± 0.19	-2.686	0.007

In Table 2, logistical results showed creatinine (CREA) (OR1.013, 95% CI: 1.006,1.021), uric acid (OR1.002, 95% CI: 1.000, 1.003), and plasma cadmium (OR 1.799, 95% CI: 1.126, 2.876) (Table 2).

**Table 2:** Results of binary logistic regression analysis of the occurrence of kidney stones and associated factors

Variables	β	SE	Wald	*P*	OR	95% CI
Constants	-1.259	0.351	12.852	0.000	0.284	
CR (μmol/L)	0.013	0.004	12.086	0.001	1.013	1.006–1.021
UA (mmol/L)	0.002	0.001	4.907	0.027	1.002	1.000–1.003
Plasma cadmium (μg/L)	0.587	0.239	6.027	0.014	1.799	1.126–2.876

The basic characteristics of the study subjects according to the quartiles of plasma cadmium are shown in Table 3. The mean plasma cadmium in the study subjects was 0.25 μg/L. The population was divided into four groups according to the three cut-off points of P25, P50, and P75 for cadmium concentration in the population. The different concentrations of cadmium in each group were associated with age, systolic blood pressure (SBP), diastolic blood pressure (DBP), triglycerides (TG), low-density lipoprotein (LDL-C), total cholesterol (TC), and the presence of plasma magnesium, plasma strontium, plasma antimony, plasma barium, and plasma lead. Total cholesterol (TC), and plasma magnesium, plasma calcium, plasma manganese, plasma copper, plasma zinc, plasma strontium, plasma antimony, plasma barium, and plasma lead were statistically different (*P* < 0.05). Compared with group Q1, group Q4 had a lower age of 58.58 ± 11.22 years old and lower low-density lipoprotein (LDL-C) amount of 3.38 ± 1.05 mmol/L. Group Q4 had higher SBP (137.63 ± 25.83 mmHg), DBP (84.00 ± 13.37 mmHg), triglyceride (TG) content (1.79 ± 2.72 mmol/L), and total cholesterol (TC) content (5.65 ± 1.18 mmol/L), as well as concentrations of other metals, including plasma magnesium (19,391.57 ± 4316.21 μg/L), plasma calcium (74,134.49 ± 13,587.99 μg/L), plasma manganese (3.96 ± 12.13 μg/L), plasma copper (955.16 ± 228.41 μg/L), plasma zinc (5930.35 ± 6607.55 μg/L), plasma strontium (32.71 ± 11.61 μg/L), plasma antimony (10.45 ± 17.33 μg/L), plasma barium (31.96 ± 18.18 μg/L), and plasma lead (10.34 ± 10.22 μg/L), as shown in Table 3.

**Table 3:** Basic characteristics of 940 participants according to quartiles of plasma cadmium

Characteristics	Quartiles of cadmium in plasma(μg/L)				F/^2^	*P*
	Q1(≤ 0.12)	Q2(0.13 ~ 0.20)	Q3(0.21 ~ 0.28)	Q4(≥ 0.29)		
Median plasma cadmium	0.10	0.16	0.23	0.41		
Gender, n (%)					3.912	0.271
Male	190(80.85)	177(75.32)	181(77.02)	192(81.70)		
Female	45(19.15)	58(24.68)	54(22.98)	43(18.30)		
Age(y)	59.20 ± 11.88	57.30 ± 11.81	56.25 ± 12.19	58.58 ± 11.22	2.939	0.032
Smoking, n (%)	81(34.45)	81(34.47)	95(40.43)	95(40.43)	3.561	0.313
Drinking, n (%)	113(48.09)	96(40.86)	107(45.53)	114(48.51)	3.515	0.319
BMI (kg/m^2^)	23.34 ± 3.45	22.70 ± 3.20	22.60 ± 3.24	22.95 ± 3.23	2.331	0.073
SBP (mmHg)	131.35 ± 21.56	132.88 ± 25.13	129.91 ± 22.71	137.63 ± 25.83	4.637	0.003
DBP (mmHg)	80.40 ± 14.23	83.00 ± 15.70	80.52 ± 13.46	84.00 ± 13.37	3.786	0.010
TG (mmol/L)	1.55 ± 1.31	1.34 ± 1.01	1.32 ± 1.02	1.79 ± 2.72	4.080	0.007
HDL-C (mmol/L)	1.70 ± 0.44	1.72 ± 0.43	1.72 ± 0.43	1.75 ± 0.45	0.602	0.614
LDL-C (mmol/L)	3.59 ± 0.99	3.45 ± 0.96	3.17 ± 1.01	3.38 ± 1.05	6.892	< 0.001
TC (mmol/L)	1.55 ± 1.13	5.46 ± 0.97	5.36 ± 1.26	5.65 ± 1.18	2.817	0.038
CREA (μmol/L)	79.00 ± 18.46	80.12 ± 21.26	84.04 ± 30.80	82.34 ± 25.84	1.984	0.115
Urea (mmol/L)	5.72 ± 1.56	5.90 ± 1.75	5.74 ± 1.75	5.95 ± 1.76	1.106	0.346
UA (mmol/L)	342.45 ± 96.51	345.33 ± 102.15	345.33 ± 95.21	349.10 ± 100.96	0.179	0.910
Plasma magnesium (μg/L)	17,438.52 ± 3053.41	18,454.79 ± 2329.49	19,013.16 ± 3144.71	19,391.57 ± 4316.21	12.449	< 0.001
Plasma calcium (μg/L)	66,775.42 ± 10,535.25	71,454.54 ± 7170.96	72,177.23 ± 9583.76	74,134.49 ± 13,587.99	17.453	< 0.001
Plasma manganese (μg/L)	1.78 ± 0.86	2.68 ± 2.18	2.96 ± 1.42	3.96 ± 12.13	57.941	< 0.001
Plasma iron (μg/L)	1128.30 ± 405.18	1205.35 ± 506.68	1184.94 ± 403.17	1268.78 ± 564.37	2.074	0.102
Plasma Copper (μg/L)	859.72 ± 203.22	893.90 ± 161.71	885.88 ± 199.42	955.16 ± 228.41	8.315	< 0.001
Plasma zinc (μg/L)	1354.25 ± 2277.92	3215.36 ± 4162.01	5430.06 ± 6265.83	5930.35 ± 6607.55	70.425	< 0.001
Plasma strontium (μg/L)	25.70 ± 9.15	30.02 ± 10.61	32.18 ± 12.18	32.71 ± 11.61	23.350	< 0.001
Plasma antimony (μg/L)	2.55 ± 4.51	3.19 ± 7.31	2.90 ± 6.29	10.45 ± 17.33	31.019	< 0.001
Plasma barium (μg/L)	20.86 ± 9.83	26.64 ± 10.75	31.54 ± 12.76	31.96 ± 18.18	45.662	< 0.001
Plasma lead (μg/L)	5.70 ± 19.40	8.87 ± 12.68	11.03 ± 14.40	10.34 ± 10.22	65.298	< 0.001

*BMI* Body mass index, *P*-values were normally distributed continuous variables tested by one-way, *ANOVA* Non-normally distributed continuous variables were tested with Kruskal–Wallis H-test. Categorical variables were tested with Pearson chi-square test

**Fig. 1:**
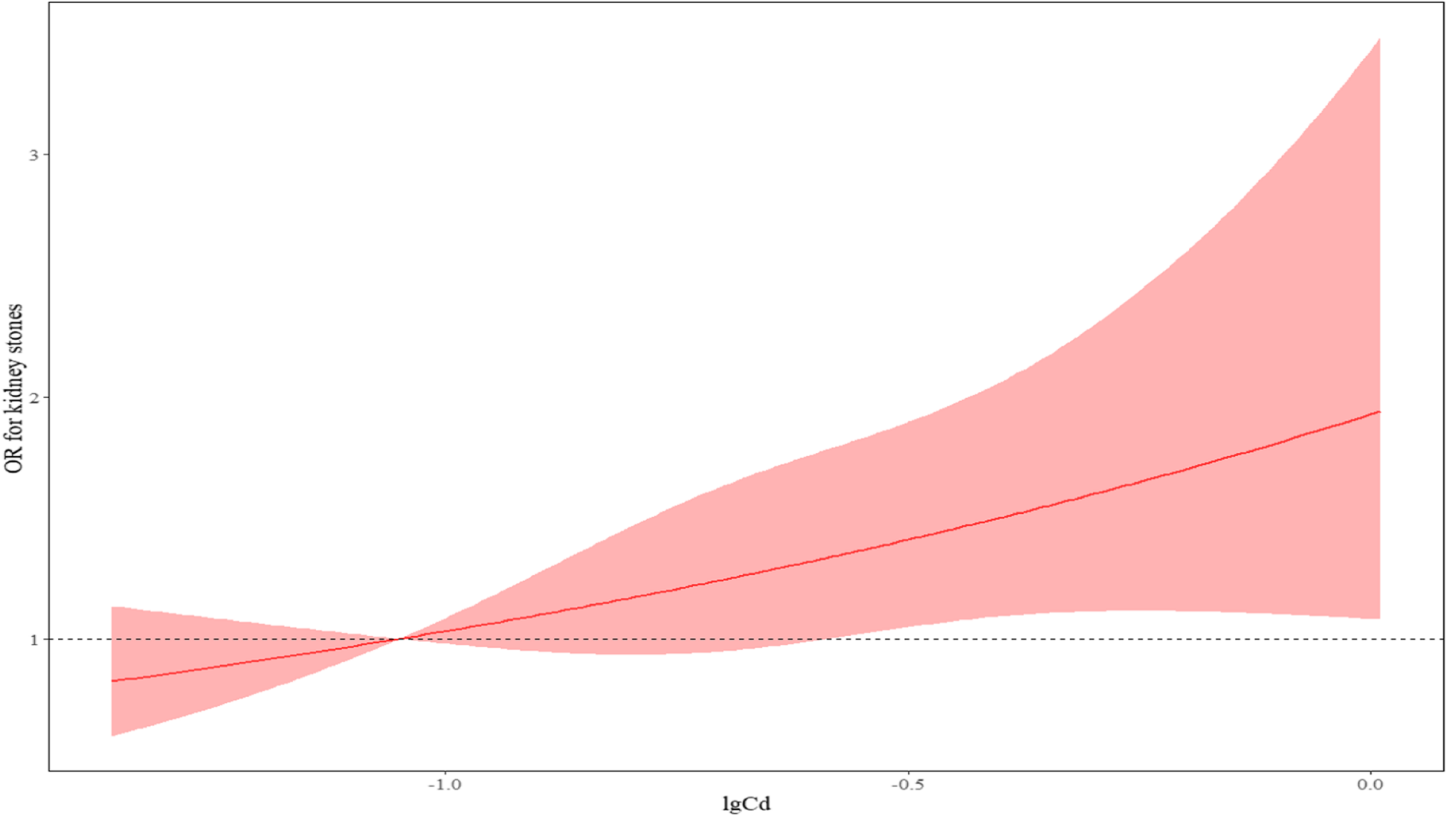
Plasma cadmium (Cd) in the total population (*n* = 940) and dose–response relationship with kidney stone; *P* = 0.039; Nonlinear *P* = 0.999

The relationship between plasma cadmium and the prevalence of kidney stones is shown in Table 4. As shown in model 1, the crude ORs (95% CIs) for kidney stones were 1.11 (95% CI, 0.77–1.59), 1.41 (95% CI, 0.98–2.02), and 1.61 (95% CI, 1.12–2.32). After including age, gender, ethnicity, marital status, occupation, education level, alcohol consumption status, smoking status, BMI, systolic blood pressure (SBP), and diastolic blood pressure (DBP) (model 2), plasma cadmium remained correlated with kidney stones (P for trend = 0.025). After multivariate correction for factors influencing kidney stones, the association between kidney stones and plasma cadmium remained after the second quartile as OR = 1.02 (95% CI, 0.70–1.48), the third quartile as OR = 1.25 (95% CI, 0.85 -1.82), and the fourth quartile as OR = 1.61 (95% CI, 1.10–2.34) (P for trend = 0.048) (model 3). In addition, as shown in Fig. 1, no non-linear relationship was found between plasma cadmium and the occurrence of kidney stones, and plasma cadmium was linearly and positively associated with the risk of kidney stone development (*P* = 0.039).

**Table 4:** Multivariable-adjusted relationship between plasma cadmium and kidney stones (*n* = 940)

	Quartiles of plasma cadmium (μg/L)				P for trend
	Q1(≤ 0.12)	Q2(0.13 ~ 0.20)	Q3(0.2 ~ 0.28)	Q4(≥ 0.29)	
Median plasma cadmium	0.10	0.16	0.23	0.41	
Model 1 (95% CI)	1(reference)	1.11(0.77,1.59)	1.41(0.98,2.02)	1.61(1.12,2.32)	0.040
Model 2 (95% CI)	1(reference)	1.10(0.76,1.59)	1.40(0.97,2.02)	1.69(1.16,2.44)	0.025
Model 3 (95% CI)	1(reference)	1.02(0.70,1.48)	1.25(0.85,1.82)	1.61(1.10,2.34)	0.048

Model 1 was the crude model. Model 2 included age (continuous data), gender (male, female), BMI (continuous data), nation (Han/Yao/Zhuang and others), marital status (unmarried and others/married), occupational (farmers/others), education level (below junior high school/junior high school and above), SBP (continuous data), DBP (continuous data), smoking status (yes/no), and drinking status (yes/no). Model 3 added creatinine (continuous data), urea (continuous data), and uric acid (continuous data) on the basis of Model 2.

## Discussion

We explored the relationship between metallic elements in blood and the risk of kidney stone development by collecting physical examination data and blood samples from rural adults in the minority concentrated areas of Guangxi, China. Creatinine (CREA), urea (UREA), and uric acid (UA) were higher in the kidney stone group than in the control group. CR, UREA, and UA are classical indicators commonly used in clinical practice to evaluate kidney function. Blood creatinine level can more accurately reflect glomerular filtration function [18]. There is increasing epidemiological evidence that blood UA levels and kidney stones are closely related and that uric acid can affect glomerular function and potentially impair tubular function [19, 20]. The values of plasma strontium, plasma antimony, plasma barium, and plasma cadmium were greater in the kidney stone group than in the control group. When renal function decreased and plasma strontium concentration increased, the underlying mechanism may be as follows: Sr^2+^ was less excreted via the kidneys. Thus, the plasma Sr^2+^ concentration in the kidney stone group was greater than that in the control group. This result was consistent with the results of previous studies [21, 22]. Antimony is a common environmental pollutant that is widely present in the natural environment, and antimony exposure can induce histopathological changes in the kidney [21–23]. Whereas epidemiological data on the effects of barium on human health are scarce, the risk of exposure to barium is higher in non-occupational populations, who are exposed mainly through drinking water and food [24]. Our study subjects were from rural areas, and none had occupational metal exposure; whether their exposure was through diet and drinking water needs to be studied next. Considering that the kidney is the main target organ for cadmium exposure [25], we performed further analysis on plasma cadmium.

In our study, systolic blood pressure (SBP), diastolic blood pressure (DBP), triglycerides (TG), total cholesterol (TC), and plasma magnesium, plasma calcium, plasma manganese, plasma copper, plasma zinc, plasma strontium, plasma antimony, plasma barium, and plasma lead were greater in the high concentration of cadmium group than in the low concentration of cadmium group, and low density lipoprotein (LDL-C) was less than that in the low concentration group. Cadmium contamination in the environment was associated with a variety of diseases, including kidney and cardiovascular system diseases. Experimental animal studies have shown that high levels of Cd exposure are associated with elevated plasma LDL, TG, and TC levels [15, 16]. Cd may damage the cardiovascular system by inhibiting biogenic amine uptake, Na^+^, K^+^-ATPase activity, and voltage-dependent Ca^2+^ channels [17]. In addition, cadmium can deactivate the vascular elasticity factor nitric oxide and disrupt endothelial homeostasis, which in turn causes cholesterol accumulation [26, 27]. However, urinary cadmium exposure is reportedly associated with lower plasma TC and LDL levels in 9-year-old Bangladeshi children [28]. The reasons for the inconsistent results may be related to different regions, age groups, and sample sizes. In addition, cadmium interferes with the function of essential minerals, such as magnesium, calcium, copper, and zinc [29]. Cadmium uptake by cells is mediated by the manganese transporter [30]. This may be the reason for the high concentrations of cadmium, magnesium, calcium, copper, zinc, manganese, and other metals. Lead is a heavy metal that accumulates in the body, especially in the bones and teeth, thereby affecting the nervous system, reproduction, and fertility and causing genotoxicity and carcinogenicity [31]. Some experimental studies suggested that lead and cadmium may have antagonistic effects [32]. However, in our study, the content of lead was higher in the high concentration group of cadmium compared with the low concentration group of cadmium, which may be due to the existence of different interactions between lead and cadmium at different concentrations. The correlation between cadmium and strontium, antimony, barium, and other metal elements are less frequently reported and need to be further studied.

Blood cadmium and urine cadmium are common indicators of internal exposure to cadmium, and blood cadmium levels reflect recent cadmium exposure [33]. In this study, blood cadmium levels were used to reflect the recent cadmium exposure of the body. Logistic regression model analysis showed that plasma cadmium levels were associated with the development of kidney stones. This was consistent with the findings of a study of 1302 Flemish people [34] and a study of workers exposed to cadmium [35]. Cadmium produces irreversible damage to complex tubules and glomeruli formed with sulfur-based proteins [36]. Blood cadmium (> 1 μg/L) is associated with higher rates of proteinuria and chronic kidney disease in the US cohort [36, 37]. In our study, plasma cadmium was at 0.26 ± 0.21 μg/L in the kidney stone group population, indicating that low doses of cadmium are still correlated with kidney injury. Therefore, the health risks associated with low levels of cadmium exposure should not be ignored. Chronic exposure to cadmium can alter the expression of fibrotic markers, activate epithelial-mesenchymal transition, and stimulate fibrotic histopathological changes [38]. The accumulation of cadmium in the body not only causes damage to the renal tubules, but also has a certain effect on glomerular function [39, 40]. Logistic regression showed that plasma cadmium was a risk factor affecting creatinine (CREA) and uric acid (UA) in vivo (*P* < 0.05), indicating an association between cadmium and impairment of renal function. The restricted cubic spline model can analyze the nonlinear relationship between the independent and dependent variables, which can reduce the bias caused by subjective classification of continuous variables. Therefore, this study presented further analysis by using a restricted cubic spline model [41]. In this study, three nodes with log-transformed plasma cadmium values, -1.05, -0.71, and -0.36, were selected because the model had the smallest value of the deficit pool information criterion when the number of nodes was at three, i.e., the model fit was optimal at this time. However, we found that a linear association existed between plasma cadmium and the risk of developing kidney stones, which was not applicable to the nonlinear model. The trend of increasing risk of developing kidney stones with increasing plasma cadmium in our study suggested that plasma cadmium may show a positive association with kidney stones.

This was a population-based study in a region with a concentration of ethnic minorities, and all study subjects were free of occupational metal exposure, which could well represent the possible dose–response relationship between plasma cadmium levels and the development of kidney stones in rural adults. The present study is a case–control study, and results can be used to speculate on the relationship between plasma cadmium and kidney stones. The conclusions drawn were consistent after adjusting for confounding factors, thereby indicating the reliability of the findings. Potential limitations of the present study were as follows. First, the relationship between dietary cadmium intake and the occurrence of kidney stones was not evaluated. However, several studies have shown that dietary intake is the main route through which cadmium enters the body and affects health [42, 43]. Studies on dietary cadmium intake and the risk of kidney stones are warranted. Second, there may be other environmental factors that influenced the occurrence of kidney stones due to cadmium exposure, and further studies are needed. Third, the sample size of the kidney stone population included in this study was small. The increased risk of kidney stones due to cadmium exposure still needs to be further confirmed in a large prospective cohort study.

## Conclusions

A positive correlation may exist between cadmium exposure and kidney stones. Increased plasma cadmium concentration may be a risk factor for the development of kidney stones.

## Data Availability

The datasets used and/or analysed during the current study available from the corresponding author on reasonable request.
